# Crohn's Disease Developing After Hirschsprung's Disease in a Child With Down Syndrome: A Rare Clinical Overlap

**DOI:** 10.7759/cureus.105497

**Published:** 2026-03-19

**Authors:** Hakar Mohammed, Anna Fursova, Dimitry Pechkurov, Farhan Abdi, Alexey Liberman

**Affiliations:** 1 Institute of Clinical Medicine, Samara State Medical University, Samara, RUS; 2 Department of Childhood Diseases, Samara State Medical University, Samara, RUS; 3 Department of Radiology, Samara Medical Radiation Center, Samara, RUS

**Keywords:** crohn disease, down syndrome, hirschsprung disease, inflammatory bowel disease, necrotizing enterocolitis, pediatric surgery, postoperative complications

## Abstract

We describe a rare pediatric case of an eight-year-old boy with Down syndrome who developed Hirschsprung's disease in the neonatal period and was later diagnosed with Crohn's disease, representing an exceptionally uncommon clinical overlap. The patient experienced a complex clinical course marked by necrotizing enterocolitis, intestinal perforation, multiple abdominal surgeries, and persistent gastrointestinal dysfunction from infancy. Despite definitive surgical management of Hirschsprung's disease, he continued to have chronic diarrhea, malabsorption, failure to thrive, and recurrent inflammatory symptoms. Further evaluation revealed markedly elevated fecal calprotectin levels and endoscopic evidence of erosive-ulcerative colitis with pseudopolyps. Radiologic, endoscopic, and histologic findings ultimately confirmed a diagnosis of Crohn's disease with ileocolonic involvement and a recurrent disease course. Initial treatment with corticosteroids and immunomodulators resulted in only partial and transient responses, and therapy was complicated by drug-induced renal impairment. The patient was subsequently escalated to biologic therapy with infliximab, achieving partial control of intestinal inflammation, alongside ongoing nutritional support with a peptide-based enteral formula. This case underscores the diagnostic and therapeutic challenges of inflammatory bowel disease in children with Down syndrome and prior Hirschsprung's disease, in whom postoperative complications and chronic enterocolitis may obscure the diagnosis. It highlights the importance of maintaining a high index of suspicion for Crohn's disease in this population and emphasizes the need for long-term surveillance and a multidisciplinary approach to management in children with complex congenital and inflammatory gastrointestinal disorders.

## Introduction

Down syndrome (DS) is a chromosomal disorder caused by trisomy 21 and is frequently associated with multisystem involvement, including characteristic phenotypic features, congenital anomalies, and immune dysregulation [1,2]. Hirschsprung's disease (HSCR) is a congenital disorder characterized by the absence of enteric ganglion cells in the distal bowel, leading to neonatal intestinal obstruction and requiring surgical intervention [3]; it is notably more frequent in children with DS [4], and patients with DS often experience delayed diagnosis due to atypical presentations [5].

Crohn's disease (CD) is a chronic inflammatory bowel disease characterized by transmural inflammation of the gastrointestinal tract, which can affect any segment and often presents with relapsing and progressive intestinal injury; it has been increasingly recognized in pediatric populations [6]. The coexistence of DS, HSCR, and CD is exceptionally rare [7], presenting unique challenges such as diagnostic uncertainty, recurrent surgical interventions, immune vulnerability, and the need to balance immunosuppressive therapy with heightened infection risk. Importantly, distinguishing CD from Hirschsprung-associated enterocolitis (HAEC) in postoperative patients can be diagnostically challenging, as both conditions may present with chronic diarrhea, abdominal distension, and systemic inflammation. This clinical overlap may delay the recognition of inflammatory bowel disease and complicate timely diagnosis and management.

We report the case of an eight-year-old Russian boy with DS, HSCR, and CD who required multiple complex surgeries and advanced immunomodulatory therapy. The concurrence of these three conditions represents one of the rarest scenarios described in pediatric practice [7], highlighting the necessity for multidisciplinary management and further investigation into shared pathophysiological mechanisms [8].

## Case presentation

An eight-year-old Russian boy with DS, born in October 2015, had a complex medical history involving multiple congenital and acquired gastrointestinal and systemic conditions. On the seventh day of life, he developed necrotizing enterocolitis complicated by transverse colon perforation and fecal peritonitis. On day nine of life, an emergency laparotomy was performed with the creation of a colostomy. The postoperative course was initially stable, and the colostomy was closed at approximately six weeks of age; however, persistent gastrointestinal dysfunction continued.

At five months of age, the patient was hospitalized with congenital heart disease, including a ventricular septal defect and patent ductus arteriosus, complicated by secondary pulmonary hypertension and heart failure. Surgical correction of both defects was performed in March 2016. The postoperative course was complicated by bowel paresis and subsequent adhesive intestinal obstruction. In April 2016, a repeat laparotomy with resection of the rectosigmoid colon and creation of a new colostomy was performed. Histopathological examination of the resected bowel demonstrated the absence of enteric ganglion cells in the submucosal and muscular layers, confirming the diagnosis of HSCR.

In 2017, ongoing malabsorption and poor weight gain prompted a duodenal biopsy, which demonstrated villous atrophy and crypt hyperplasia consistent with secondary enteropathy. In 2018, colostomy stenosis developed; dilation attempts were unsuccessful, and the patient required colonic resection with colorectal anastomosis and appendectomy. The postoperative course was complicated by an anastomotic leak, requiring relaparotomy and creation of an ileostomy for fecal diversion.

Throughout 2019, the patient exhibited persistent failure to thrive, weight loss, and gastroesophageal reflux disease, requiring nutritional support. Given recurrent gastrointestinal symptoms and obstruction, the patient was evaluated for cystic fibrosis, which was ruled out by normal sweat chloride testing and a negative CFTR mutation panel.

In 2020, a colonoscopy with biopsy demonstrated erosive-ulcerative colitis with pseudopolyps, predominantly involving the ileocolonic region. Fecal calprotectin was markedly elevated at 1,800 μg/g. Treatment with prednisolone and mercaptopurine resulted in transient clinical improvement, with frequent relapses following corticosteroid taper.

In 2021, a follow-up evaluation demonstrated persistent erosive-ulcerative inflammatory changes despite immunosuppressive therapy. Reconstructive surgery was subsequently performed, including the formation of a coloanal anastomosis with closure of the ileostomy. The postoperative course was complicated by persistent diarrhea and fecal incontinence.

**Figure 1:**
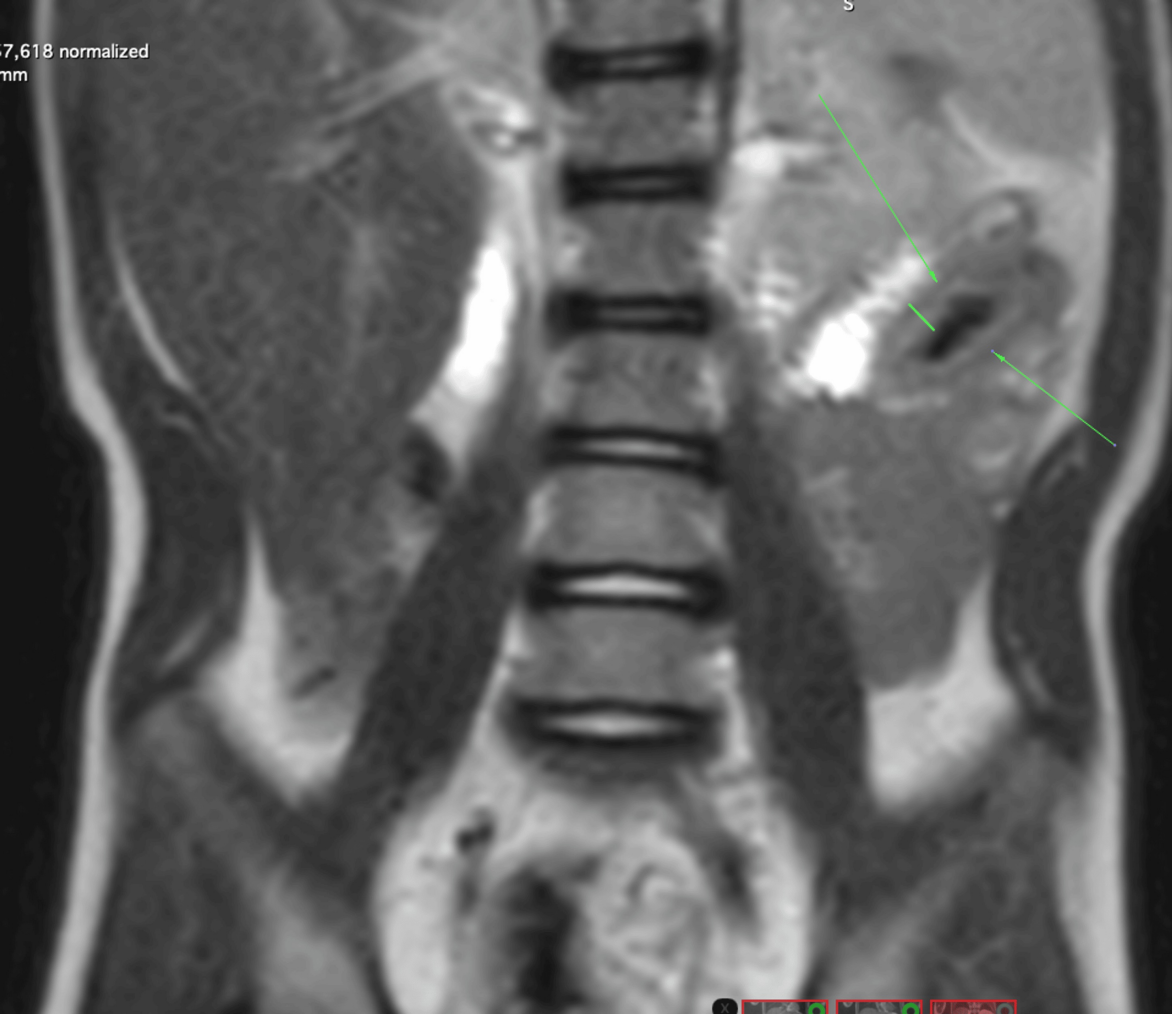
Coronal abdominal magnetic resonance imaging demonstrating bowel wall thickening Coronal T2-weighted magnetic resonance imaging of the abdomen demonstrating segmental bowel wall thickening with a cobblestone appearance, consistent with inflammatory bowel disease-related changes (arrows).

**Figure 2:**
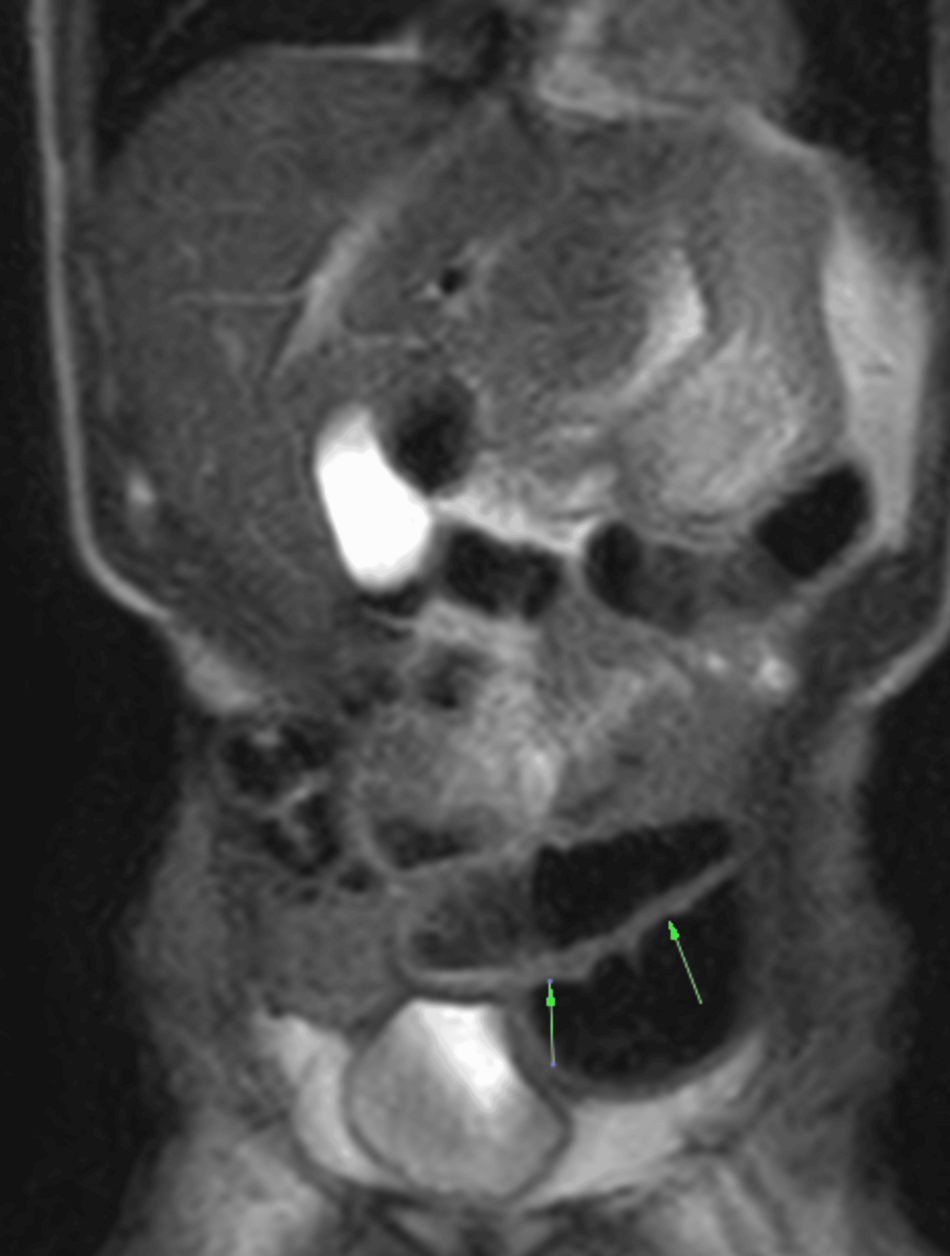
Coronal abdominal magnetic resonance imaging showing extensive ileocolonic involvement Coronal magnetic resonance image demonstrating extensive ileocolonic involvement with mural thickening and luminal narrowing, consistent with active inflammatory bowel disease (arrows).

In 2022, abdominal magnetic resonance imaging demonstrated extensive segmental bowel wall thickening with a cobblestone appearance, consistent with inflammatory bowel disease (Figures 1, 2).

Histopathology demonstrated focal transmural inflammation with chronic active colitis, consistent with CD. In conjunction with clinical presentation, endoscopic findings, and imaging, these findings confirmed CD with ileocolonic involvement and a recurrent inflammatory course.

Induction therapy with infliximab was initiated. For maintenance therapy, the patient was treated with prednisolone and azathioprine; azathioprine was discontinued due to drug-induced tubulointerstitial nephritis, associated with a decline in estimated glomerular filtration rate to 79.5 mL/min/1.73 m² (stage 2 chronic kidney disease). In 2023, recurrent fecal incontinence prompted posterior sphincter levatorplasty with puborectal muscle reconstruction, resulting in partial improvement.

At present, the patient is maintained on infliximab with partial control of intestinal inflammation, along with a peptide-based enteral formula (Peptamen Junior) to address malabsorption and protein-energy malnutrition. Ongoing comorbidities include chronic gastroduodenitis, small intestinal bacterial overgrowth, and allergic enteropathy. Neurological manifestations include global developmental delay, diffuse hypotonia, speech delay, and mixed-type encephalopathy related to DS. Endocrine evaluation revealed subclinical hypothyroidism. The patient also has a history of recurrent *Clostridium* infections and immune dysfunction within the spectrum of DS. Cardiac status remains stable following surgical correction. He is currently managed with a multidisciplinary supportive care approach addressing chronic intestinal disease, nutritional deficits, renal impairment, and immunological vulnerability.

A comprehensive chronological summary of the patient’s clinical course from 2015 to 2023 is presented in Table 1.

**Table 1:** Chronological timeline summarizing the patient’s major clinical events, surgical interventions, diagnostic findings, and therapeutic modifications from 2015 to 2023 VSD: ventricular septal defect; PDA: patent ductus arteriosus; GERD: gastroesophageal reflux disease; eGFR: estimated glomerular filtration rate; MRI: magnetic resonance imaging

Year	Clinical event	Intervention performed	Key diagnostic findings	Outcome/diagnosis
2015	Necrotizing enterocolitis (NEC) with transverse colon perforation and fecal peritonitis	Emergency laparotomy + colostomy creation	Clinical presentation of NEC; intraoperative findings of colon perforation and peritonitis	Stabilization; colostomy closed at ~6 weeks
2016	Congenital heart disease (VSD, PDA) with pulmonary hypertension and heart failure	Surgical VSD and PDA repair	Cardiac imaging confirming VSD and PDA; evidence of pulmonary hypertension	Cardiac stabilization
	Adhesive intestinal obstruction	Repeat laparotomy + rectosigmoid resection + new colostomy	Histopathology: absence of enteric ganglion cells in submucosal and muscular layers	Histology confirmed Hirschsprung's disease
2017	Malabsorption, poor weight gain	Duodenal biopsy	Duodenal biopsy: villous atrophy and crypt hyperplasia	Secondary enteropathy
2018	Colostomy stenosis	Colonic resection + colorectal anastomosis + appendectomy	Failed dilation attempts; clinical stenosis	Complicated by anastomotic leak
	Anastomotic leak	Relaparotomy + ileostomy creation	Clinical and intraoperative evidence of anastomotic leak	Diversion achieved
2019	Persistent failure to thrive, weight loss, GERD	Nutritional support	Normal sweat chloride test; negative CFTR mutation panel (ruled out cystic fibrosis)	Ongoing growth impairment
2020	Inflammatory colitis	Prednisolone + mercaptopurine	Fecal calprotectin 1,800 μg/g; colonoscopy with biopsy: erosive-ulcerative colitis with pseudopolyps, ileocolonic involvement	Steroid-dependent disease; frequent relapses
2021	Refractory colitis with persistent inflammation	Coloanal anastomosis + ileostomy closure	Persistent erosive-ulcerative inflammatory changes on follow-up evaluation	Persistent diarrhea and fecal incontinence
2022	Established inflammatory bowel disease with progression	Diagnostic evaluation completed	Abdominal MRI: extensive segmental bowel wall thickening with cobblestone appearance; histopathology: focal transmural inflammation with chronic active colitis	Crohn's disease diagnosed (ileocolonic, inflammatory type)
2023	Recurrent fecal incontinence	Posterior sphincter levatorplasty with puborectal reconstruction	Clinical assessment of sphincter dysfunction	Partial improvement
	Azathioprine-induced tubulointerstitial nephritis (eGFR 79.5 mL/min/1.73 m²)	Azathioprine discontinued	eGFR decline to 79.5 mL/min/1.73 m²; drug-induced tubulointerstitial nephritis	Stage 2 chronic kidney disease
	Ongoing Crohn's disease activity	Infliximab therapy + peptide-based enteral nutrition	Clinical and biochemical evidence of active inflammation	Partial control of inflammation

## Discussion

DS is the most common chromosomal disorder worldwide, with an estimated prevalence of one in 1,000 to one in 1,100 live births, and is frequently associated with congenital anomalies, immune dysfunction, and gastrointestinal pathology [1,2]. Among these, HSCR, a congenital aganglionosis of the distal bowel, occurs in approximately one in 5,000 live births and is significantly more common in children with DS, affecting about 2%-3% of patients based on population-level data [3,4]. CD, a chronic inflammatory bowel disease, has a prevalence of 58 per 100,000 and is quite rare in association with DS [6]. The coexistence of DS, HSCR, and CD in a single patient represents a rare and clinically challenging situation.

Each disorder independently contributes to significant morbidity in children, but their combination creates unique challenges for both diagnosis and management. Patients with DS often experience delayed diagnosis of HSCR compared with those without DS [5]. Although CD is primarily diagnosed in adults, it should still be considered in DS patients who present with persistent or unexplained gastrointestinal symptoms [7]. The overlap between these conditions may not be coincidental. Recent molecular studies have shown that HSCR and CD share overlapping immune-inflammatory pathways, suggesting that their rare coexistence may have a biologically plausible mechanistic basis [8].

Children with HSCR have an increased lifetime risk of developing inflammatory bowel disease, particularly CD, occurring in more than 2% of patients with HSCR [9]. In postoperative HSCR patients, chronic diarrhea, recurrent HAEC, and mucosal inflammation can clinically mimic CD, often delaying the correct diagnosis [10].

In such complex cases, biologic therapy with anti-TNF agents such as infliximab is effective for inducing and maintaining remission in pediatric CD when conventional immunosuppression is insufficient [11]. Finally, intensive nutritional support (including exclusive or supplemental enteral nutrition) is critical in these children because pediatric CD is strongly associated with malnutrition and growth failure, and nutrition-focused therapy improves mucosal healing, growth, and overall disease control [12].

## Conclusions

This case underscores the importance of a multidisciplinary approach in children with overlapping congenital and inflammatory gastrointestinal disorders, such as DS, HSCR, and CD. Long-term surveillance, individualized surgical planning, and tailored medical therapy are crucial to address complex surgical complications and diagnostic challenges, including histological difficulties. Recognition of these key clinical indicators, along with further study of shared pathophysiological mechanisms, may refine treatment strategies and improve outcomes in similar patients.
